# Prospective comparison of ligation and bipolar cautery technique in non-scalpel vasectomy

**DOI:** 10.1590/S1677-5538.IBJU.2014.0356

**Published:** 2015

**Authors:** Muammer Altok, Ali Feyzullah Şahin, Rauf Taner Divrik, Ümit Yildirim, Ferruh Zorlu

**Affiliations:** 1 Department of Urology, Süleyman Demirel University, Faculty of Medicine, Isparta, Turkey; 2 Department of Urology, Şifa University, Faculty of Medicine, Izmir, Turkey; 3 Department of Urology, Gazi Hospital, Izmir, Turkey; 4 Department of Urology, M.H. Tepecik Research and Education Hospital, Izmir, Turkey

**Keywords:** Cautery, Ligation, Vasectomy

## Abstract

**Objectives:**

There is no trial comparing bipolar cautery and ligation for occlusion of vas in non-scalpel vasectomy. This study aimed to compare the effectiveness of these vasectomy occlusion techniques.

**Materials and Methods:**

Between January 2002-June 2009, patients were allocated in alternate order. We recruited 100 cases in cautery group and 100 cases in ligation group. Non-scalpel approach was performed during vasectomy and fascial interposition was performed in all cases. First semen analysis was done 3 months after vasectomy. Vasectomy success was defined as azoospermia or non-motile sperm lower than 100.000/mL.

**Results:**

Four patients from the cautery group were switched to the ligation group due to technical problem of cautery device. Thus, data of 96 patients as cautery group and 104 patients as ligation group were evaluated. After vasectomy, semen analyses were obtained from 59 of 96 (61.5%) patients in cautery group and to 66 of 104 (63.5%) patients in ligation group. There was no statistical significant difference between the two groups in terms of the success of vasectomy (p=0.863).

**Conclusion:**

Although bipolar cautery technique is safe, effective and feasible in non-scalpel vasectomy, it has no superiority to ligation. There was no statistically significant difference in terms of the success and complications between the two groups.

## INTRODUCTION

Vasectomy is a popular and effective family planning method today. Vasectomy has two main steps: exposing the vas out of the scrotum and occluding the vas. Conventional, non-scalpel, and percutaneous methods are used for the isolation of the vas (1, 2). The non-scalpel vasectomy technique, which is commonly used for the isolation of the vas, was described in 1974 in China by Dr. Li Shunqiang (3).

There are various methods to occlude the vas when performing a vasectomy, such as the division and excision of a segment, the ligation of the vas with metal clips or suture materials, cauterization of the mucosa of the vas lumen, fascial interposition, and a folding back of the divided vas. A fascial interposition (FI) is the only vasectomy occlusion that was well evaluated in a randomized trial. (4) Sokal et al. (5) demonstrated that adding an FI to the ligation with the suture material and the excision of a 1cm segment significantly reduced the failures by about half based on the semen analysis, from 12.7% to 5.9%.

In the United States, the cautery is the most commonly used method for the occlusion of the vas (6). However, in low-income countries, the ligation and excision is the most widely used occlusion technique (7). Comparative studies suggested that intraluminal cauterization of the ends of the vas, is more effective than ligation (8–10). However, there is no randomized trial comparing these two approaches, yet (4).

Intraluminal cauterization was the main technique of the trials when an electro-cautery or thermal cautery was used (7, 11, 12). There is no prospective comparative trial with a bipolar cautery and ligation for the occlusion of the vas. Our study aimed to compare the effectiveness of these techniques when both are combined with FI.

## MATERIALS AND METHODS

From January 2002 to June 2009, patients were divided and given a number in order of their applications. Odd numbers were given to patients who were included in the ligation group, and even numbers were given to patients who were included in the cautery group. The study was planned with 100 cases in each group. The information about vasectomies was given to the couples before the operation, and their informed consent was obtained. Patients who chose the vasectomy as a family planning method, who agreed to participate in the study, and who did not have a history of a vasectomy, scrotal, inguinal, or pelvic surgeries were included in the study.

**Figure 1:**
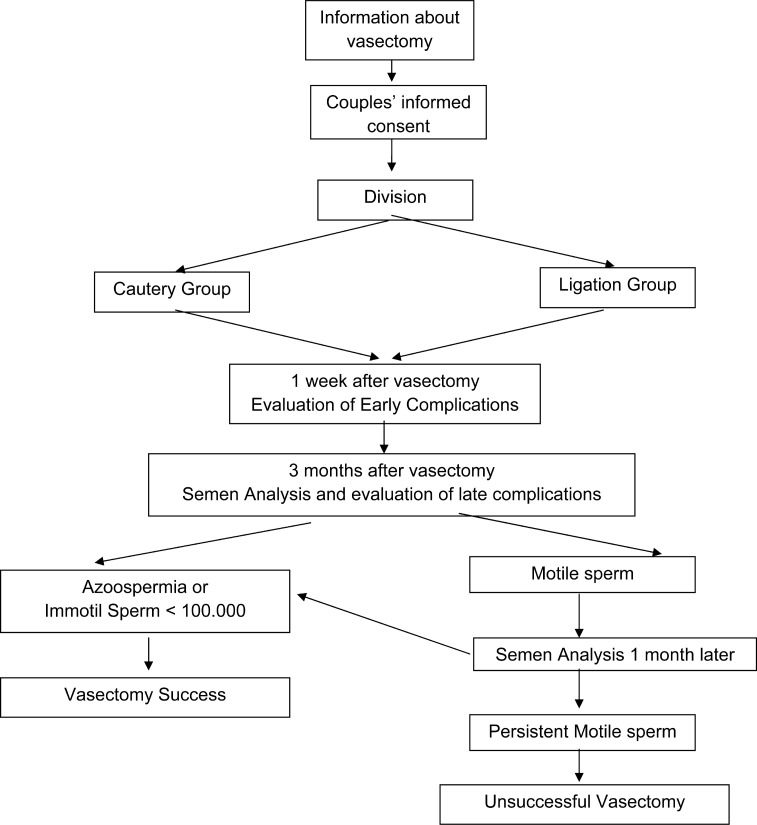
Study Diagram.

The procedure was performed by three experienced surgeons in the Urology Department of Tepecik Research and Education Hospital, a specialized urology clinic. After a local anesthesia was given, the isolation of the vas was performed using the non-scalpel approach (3, 13). In the cautery group, approximately 1cm segment of the vas was excised, and the extremities of both ends of the divided vas were cauterized using a bipolar cautery. In this technique, unlike the classic intraluminal cauterization, only the mucosal mouth of the lumen was sandwiched and fulgurated/cauterized between the two tips of the bipolar cautery from different sides until the lumen was closed. Bipolar cautery was not inserted into the lumen of the vas. In the ligation group approximately 1cm segment of the vas was excised and both ends of the vas were ligated using a silk 3/0. FI was performed on the prostatic end of the vas in all of the patients in both groups (3, 13). Patients were evaluated for complications one week after vasectomy. The first semen analysis was done three months after vasectomy. Vasectomy success was defined as azoospermia or presence of non-motile sperm lower than 100.000/mL (12, 14). Semen analysis was repeated one month later in the patients with motile sperm. If motile sperm was still detected in a repeated semen analysis, these cases were classified as an unsuccessful vasectomy (Figure-1).

The study was approved by the Local Ethics Committee. The chi-squared test was used to assess the differences between the study groups. P value <0.05 was considered significant.

## RESULTS

Four patients from the cautery group were switched to the ligation group due to a malfunction of the cautery device. Thus, the data of 96 patients in the bipolar cautery group and 104 patients in the ligation group were evaluated. The characteristics of the patients, including age, duration of marriage, number of children, and education level were similar in both groups (Table-1). Vasectomy was performed without any problem in all of the patients.

**Table 1:** Characteristics of patients.

Characteristics (Mean ± SD)		Cautery Group	Ligation Group	p
N		96	104	
Age (range)		40.6 ± 5.7 (27–58)	40.8 ± 5.8 (25–55)	0.806
Years of marriage (range)		15.3 ± 5.6 (2–27)	16.0 ± 5.5 (4–32)	0.373
Number of children (range)		2.5 ± 0.9 (1–6)	2.5 ± 0.9 (1–7)	0.999
**Educational level n (%)**				0.937
	No education	0 (0)	1 (1.0)	
	Primary	64 (66.7)	68 (65.4)	
	High school	22 (22.9)	24 (23.1)	
	University	10 (10.4)	11 (10.6)	

**SD =** Standard Deviation

After vasectomy, semen analyses were obtained from 59 of the 96 (61.5%) patients in the cautery group and from 66 of the 104 (63.5%) patients in the ligation group. The proportion of patients with successful occlusions was similar in the cautery (55/59, 93.2%) and the ligation (61/66, 92.4%) groups (p=0.863; Table-2). All of the patients in the cautery group who were considered successful were azoospermic. However, four patients had rare non-motile sperm in the ligation group (Table-2). The proportion of patients with presumed recanalization was similar in both groups (7.8% vs. 7.6%, Table-2).

**Table 2:** Semen analysis results.

Group	N	Azoospermia or Immotil Sperm<100.000	Motil Sperm
**Cautery**	59	55 (93.2%)	4 (6.8%)
**Ligation**	66	61 (92.4%)	5 (7.6%)
**Total**	125	116 (92.8%)	9 (7.2%)

Chi-square test p:0.863

We phoned the 75 patients who did not return for semen analysis after vasectomy and reached 38 patients (21 from cautery group and 17 from ligation group). No pregnancies were reported. Considering this additional information, the success rate could be estimated as 95% (76/80) in the cautery group and 93.9% (78/83) in the ligation group (p=0.76; Table-3).

**Table 3:** If avoiding pregnancy is accepted as vasectomy success.

Group	Azoospermia or Immotil Sperm<100.000	Pregnancy (-)
Cautery	55 (93.2%)	76 (95%)
Ligation	61 (92.4%)	78 (93.9%)

Chi-square test p:0.76

Intraoperative complications were not observed in the bipolar cautery group. However, a vasovagal reflex occurred in one patient in the ligation group. Postoperative hematomas were observed in three (3.1%) cases in the cautery group, one with epididymitis requiring antibiotics and one requiring drainage, and none were observed in the ligation group (p=0.07). In the ligation group, no surgical complications were observed, but three patients had psychological problems associated with the vasectomy and emotional distress was observed in these patients (2.8%).

## DISCUSSION

Very few studies that address vasectomy occlusion techniques are prospective and even fewer studies are randomized or quasi-randomized (4, 10, 12). The review of the failure rates for the intraluminal cautery technique with FI ranged from 0% to 0.55% (12). According to the American Urological Association (AUA) (12) and the European Association of Urology (EAU) (14) vasectomy guidelines, the most effective vas occlusion technique is the intraluminal mucosal cauterization with FI; however, without FI is also likely to be consistently effective. In our study, we used a bipolar cautery and occluded only the mucosal mouth of the lumen with cauterization instead of intraluminal cauterization. To our knowledge, this is the first prospective trial for a bipolar cautery with FI in a non-scalpel vasectomy. The occlusive failure rate (presumed recanalization) of 6.8% in our study was much higher than those reported in the other studies about intraluminal cautery. In the bipolar technique, unlike the classic intraluminal mucosal cautery method, only the mucosal mouth of the vas was occluded until it seems to be closed. We considered that the results were inadequate because of the insufficient necrosis and subsequent occlusion. The occlusive failure of over 1% is considered unacceptable according to the AUA guidelines (12).

Failure rates for the ligation technique with FI ranged from 0% to 5.85% (12). The only randomized controlled trial that evaluated this technique was reported by Sokal et al. (5). Their reported a failure rate of 5.85%. Our failure rate was also higher in our study (7.6%) in the ligation group.

In our study, vasectomy success was not statistically or significantly different between the study groups. However, our study lacked the statistical power to observe a small difference between the groups. If the bipolar cautery technique with FI had been as effective as the other cautery technique reported in the literature (under 1%), the difference with the ligation group (7.6%) would have been highly significant.

Of all the patients, 75 (37 (38.5%) from the cautery group and 38 (36.5%) from the ligation group) patients did not return for semen analysis after vasectomy. Thomas et al. evaluated the compliance of 1,892 patients after vasectomy and reported that 34% of patients did not return after the procedure (15). Sheynkin et al. also evaluated the compliance of 214 vasectomy-performed patients and detected that 46.2% of the patients did not return for the semen analysis (16). They reported that the rate of noncompliance was independently higher in men with four or more children, smokers, and those with a lower education level (16). These results are similar to our study. We phoned these 75 patients and reached 38 patients (21 from the cautery group and 17 from the ligation group). The main factor of noncompliance was the religious and cultural effect of masturbation. Pregnancy was not detected in any of these patients. If pregnancy was accepted as a vasectomy success, the failure rate of our study would be a little lower (Table-3). In this case, the failure rate (6.1%) in the ligation group of our study would be similar to the literature (5).

In regard to complications, there was no statistically significant difference between the two groups in our study, and the rate of postoperative surgical complications in the cautery group (3.1%) could be attributed to chance or to a lack of statistical power. However, all of the hematomas were related to the vas isolation and not to the occlusion technique. Overall, the rate of hematomas (1.5%, 3/200) in our study is in the range of 1%-2%, which is considered acceptable by the AUA (12).

Although the bipolar cautery technique combined with FI appears to be as safe as the ligation with FI as an occlusion vasectomy technique, the failure rate was much higher than the intraluminal mucosal cautery techniques. In light of these results, we no longer perform bipolar cautery in our clinic and we are considering alternative occlusion techniques to the ligation and FI to improve our occlusion effectiveness.
